# Outcomes of Neonatal Resuscitation With and Without an Intact Umbilical Cord: A Meta-Analysis

**DOI:** 10.7759/cureus.44449

**Published:** 2023-08-31

**Authors:** Santosh Avinash B, Suresh Babu Mendu, Paramesh Pandala, Rakesh Kotha, Venkata Narayana Yerraguntla

**Affiliations:** 1 Department of Pediatrics Intensive Care, Osmania Medical College, Hyderabad, IND; 2 Department of Pediatrics, Government Medical College, Siddipet, Siddipet, IND; 3 Department of Pediatrics, Government Medical College, Jangaon, Hyderabad, IND; 4 Department of Neonatology, Osmania Medical College, Hyderabad, IND; 5 Department of Neonatology, Niloufer Hospital, Hyderabad, IND

**Keywords:** neonates, apgar score, umbilical cord, neonatal resuscitation, meta analysis

## Abstract

Around the world, very few babies require a more intensive resuscitative effort for stabilization. The optimal timing of an intact umbilical cord to help with resuscitation is controversial. Our objective in the review is to compare the outcomes of neonatal resuscitation with and without an intact umbilical cord. A search of six electronic database libraries was explored for data released between 2014 and 2023. A manual search of secondary references in relevant studies was also performed. Studies focused only on randomized controlled trials comparing the outcomes of neonatal resuscitation with and without an intact umbilical cord at any gestational age. Two reviewers retrieved data for relevant outcomes and independently evaluated trial quality and eligibility. Mortality rate and APGAR (appearance, pulse, grimace, activity, and respiration) scores were noted as common in the two studies. Four randomized control trials were assessed for the impact of delayed cord clamping on neonates. One study focused on neurodevelopmental outcomes and noted significant improvement. Other studies noted delayed clamping as beneficial for improving oxygen saturation, APGAR score, and mortality rate. The meta-analysis included three controlled trials with a total of 528 babies and tested the effects of clamping the umbilical cord either late (n = 264) or early (n = 264). The heterogeneity of mortality and APGAR score at 5 minutes were not significant, which may be because only two studies of each case were available to compare. We concluded that very few studies are available to identify a significant impact of delayed cord clamping in neonates. However, delayed clamping for up to 5 minutes is noted as beneficial to the newborn.

## Introduction and background

The umbilical cord's clamping and cutting during delivery is one of the oldest human interventions. Despite this, there is still debate over when to cut the umbilical cord [1]. Over the past 10 years, randomized trials have shown that delayed clamping is beneficial for both term and preterm newborn children, even though the majority of maternity units practice early clamping (5-15 s) [2]. Around the world, 1% of babies require a more intensive resuscitative effort for stabilization, while up to 10% of newborns need therapies to ease the transition to extrauterine life [3]. Because of the physiological change from prenatal to neonatal life, resuscitation at the time of delivery is unique from all other resuscitation [4]. Clipping the umbilical cord before breathing increases systemic blood pressure due to loss of low-resistance placental circuit and high pulmonary vascular resistance, preventing left-to-right flow and low cardiac output [5,6]. Physiological studies showed that DCC (delayed cord clamping) causes transient changes in cardiac and hematological indices but no need for special care for polycythemia, jaundice, or respiratory distress [7-10].

Delaying umbilical cord clamping for 1-2 minutes, if possible, is strongly advised by several national and international practice standards [3,11]. Although late clamping is noted as helpful for neonatal, meta-analysis of the literature on outcomes of neonatal resuscitation with and without an intact umbilical cord is lacking. Thus this review will help to understand the impact of late clamping on neonatal resuscitation.

## Review

Method

This analysis aimed to evaluate differences in the outcomes of neonatal resuscitation with and without an intact umbilical cord. It included studies that clearly mentioned randomized clinical trials where resuscitation was initiated with and without an intact umbilical cord. We registered with ID 444835 under PROSPERO (International Prospective Register of Systematic Reviews).

Literature search strategy

We constructed a search strategy using a PICO (population, intervention, control, and outcomes) framework (Table 1). We looked for appropriate published studies using a variety of sources in accordance with the Cochrane Collaboration Guidelines for systematic reviews [12].

**Table 1:** PICO Framework Used in Our Review APGAR: appearance, pulse, grimace, activity, and respiration

PICO Framework	
P (Population)	Neonate
I (Intervention)	Delayed Cord Clamping
C (Control)	Early Cord Clamping
O (Out Comes)	Mortality and 5-minute APGAR Score
S (Study Type)	Randomized Controlled Trails

The search comprised abstracting, referencing, and indexing electronic database libraries released between 2014 and 2023. Six databases, including the Cochrane Central Register of Controlled Trials (CENTRAL), Pubmed, MEDLINE, EMBASE, clinicaltrials.gov, and CINHAL databases, were included. This survey included all studies comparing early and delayed umbilical cord clamping. Additionally, manual searches were done by looking through the reference lists of the studies that were included.

We took text keywords from our topic, and from the keywords we identified Medical Subject Headings (MeSH). A draft search strategy was developed for MEDLINE and other databases using MeSH and free text keywords related to neonatal intact umbilical cord resuscitation and DCC. Reviews published in the English language will be taken. We used Boolean operators “OR” and “AND” parentheses () and field codes used for both text and abstracts (Text/Abstract). The search strategy will be adapted and modified accordingly for use in other databases. We searched the sentences: (“neonatal intact cord resuscitation*”), (“delayed umbilical cord in a neonate*”) ("intact cord resuscitation*") or (Neonatal outcomes*).

Keywords used were (Neonate or Newborn ) OR resuscitation, (Neonate or Newborn) OR (“intact umbilical cord resuscitation*”), (Neonate or Newborn) OR (“Delayed Cord Clamping*”), (Neonate or Newborn) OR (“intact umbilical cord resuscitation” OR “Delayed Cord Clamping*) OR (Randomised or Randomized) OR (Clinical trials*) OR (Term Neonate* OR Preterm neonate*) (Neonate or Newborn) AND (“intact umbilical cord resuscitation” OR “Delayed Cord Clamping*) AND (Randomised or Randomized OR Clinical trails*) AND (Term Neonate* OR Preterm neonate*).

Identification of Study

At least two review authors separately evaluated whether it was suitable to include the full texts of potential research. Citations were checked using titles and abstracts. Duplicate citations were deleted, and every citation has been exported to EndNote. Discrepancies were made clear in order to achieve unity by the third reviewer.

Data Extraction

The following were considered inclusion criteria for the present study: 1. controlled trials; 2. studies comparing intact cord resuscitation and without intact cord resuscitation; 3. infant delivery by either vaginally or by cesarean section; 4. any gestational age of the mother; 5. original data on at least one of our interest outcomes. We used very restrictive selection criteria, such as intact cord resuscitation and randomized controlled studies. Hence, the number of available studies is very small. This is the major limitation of our study.

Exclusion criteria were fetuses with known congenital malformations, mothers with multiple pregnancies, and patients who withdrew from the study.

Meta-analysis

Revman Web (Cochrane Collaboration, Oxford, UK) was utilized for the meta-analysis. The two reviewers duplicated the data entry into RevMan. We computed the weighted mean difference (WMD) for continuous variables using the mean and standard deviation provided in the initial trials. Using fixed-effects models, estimates of the pooled outcomes and their 95% confidence intervals (CIs) were computed. We further used the 2-test for significance to evaluate trial heterogeneity. I^2^ values greater than 50%, which indicate considerable heterogeneity between studies, were used to produce pooled estimates based on random-effects models.

**Figure 1:**
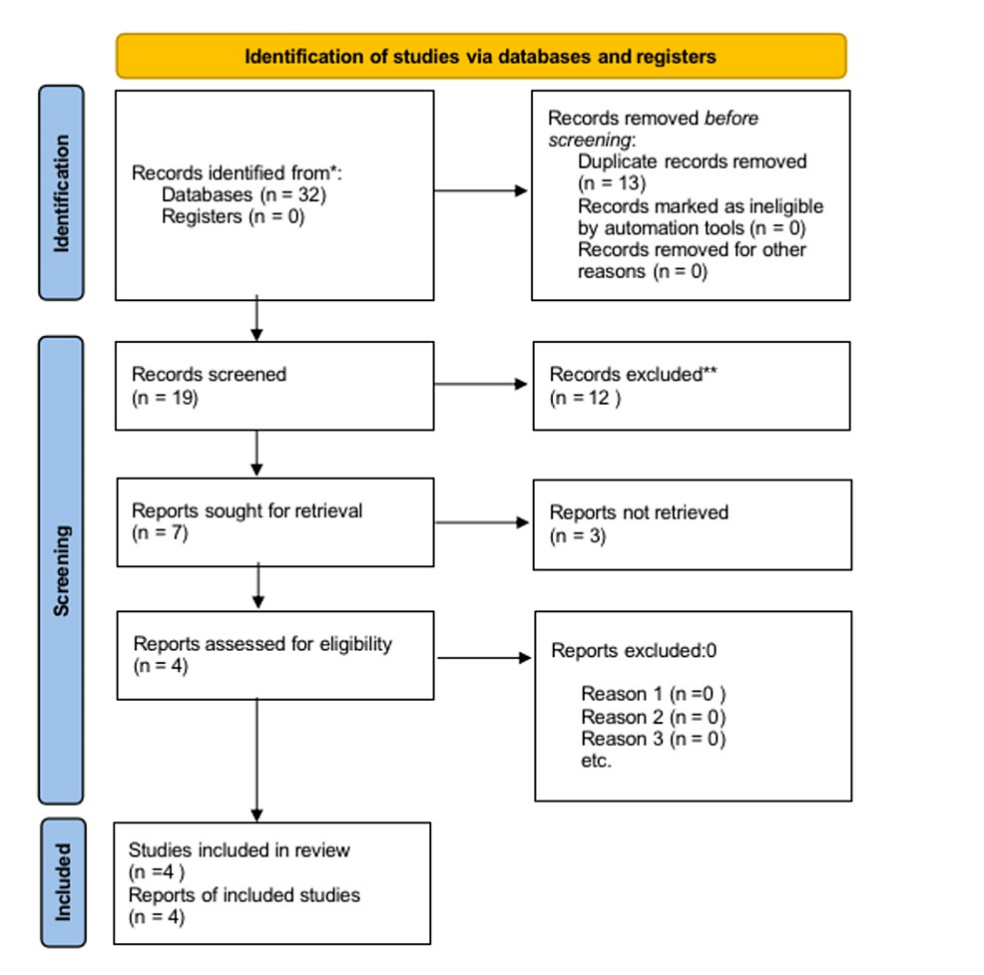
PRISMA Flow Diagram PRISMA: Preferred Reporting Items for Systematic Reviews and Meta-Analyses

Studies showing the impact of clamping on the resuscitation of neonates were screened. A total of 32 potentially eligible research studies were reviewed. Thirteen articles were noted as duplicates and eliminated from the study, and 19 studies were screened further by using titles and abstracts. Twelve studies do not meet the exact inclusion criteria. Only seven studies were found suitable and included for a thorough analysis of full-text data. Out of them, only three studies were noted as suitable to include in the present review based on inclusion criteria (Figure 1). Finally, there are only four studies listed (Table 2).

**Table 2:** Randomized Controlled Trials (N =4) Comparing Early vs. Late Cord Clamping in Neonatal Required Resuscitation at Birth DCC: delayed cord clamping

Name of the Study	Question Type	Patient, Population, Problem	Intervention or Exposure	Comparison of Control	Outcome Measures	Study Design
Duley et al., 2017 [13]	Therapy	Gestational age < 32 weeks, Resuscitation	EC <20s DC< 120s	Standard care	EC - 11.1% mortality DC - 5.2%	Randomized controlled trial- parallel-group study
Andersson et al., 2019 [14]	Therapy	Gestational age ≥ 33 weeks, Resuscitation	Early cord clamping < 60 seconds Delayed cord clamping > 180 s	Standard care	DCC - absolute risk reduction (56% higher) Apgar score (10% higher) No mortality (3.1% higher)	Randomized controlled trial- parallel-group study
Isacson et al., 2021 [15]	The prognosis (Forecast)	Gestational age between 33 and 41 weeks, Resuscitation	Early cord clamping < 60 seconds Delayed cord clamping > 180 s	Standard care	Development for age Z-score (DAZ) was significantly higher for Neurodevelopmental outcomes (p=0.04)	Randomized clinical trial
Katheria et al., 2017 [16]	Therapy	Gestational age ≥37 weeks, Resuscitation	Early cord clamping < 60s Delayed cord clamping > 300	Standard care	Greater cerebral oxygenation and blood pressure (not statistically significant)	Randomized clinical trial

Results

Three of the four studies, with a total of 528 infants, examined the effects of cord clamping either late (n = 264) or early (n = 264). Because there are few clinical trials, we did not perform subgroup analysis. According to the outcome of the studies, we divided the studies into two groups (Table 3, Table 4). The heterogeneity of the two groups was low because the number of studies was very small, the point estimates of the studies were similar, and the CIs were similar. Because of the very small number (n = 2 each) of available studies, the power to identify heterogeneity was also low.

**Table 3:** Comparative Study of Mortality DCC: delayed cord clamping, ECC: early cord clamping

S.N.	Authors	Number of Neonatal	ECC (Numbers)	DCC (Numbers)	P value
1	Duley et al., 2017 [13]	135	11.1% (14)	5.2% (7)	<0.05
2	Andersson et al., 2019 [14]	99	3.1% (3)	0 (0)	<0.07

**Table 4:** Comparative Study of Good APGAR Score (>7) at 5 Minutes APGAR: appearance, pulse, grimace, activity, and respiration, DCC: delayed cord clamping, ECC: early cord clamping

S.N.	Authors	Number of Neonatal	DCC	ECC	P value
1	Andersson et al., 2019 [14]	100	83	73	<0.07
2	Katheria et al., 2017 [16]	30	22	22	0.16

**Figure 2:**
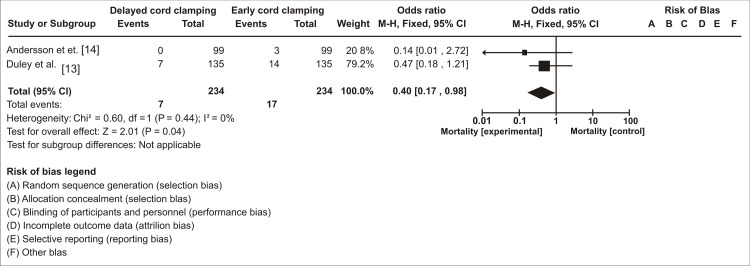
Rate of Mortality among Infants With Delayed Cord Clamping (DCC) Relative to Early Cord Clamping (ECC)

When comparing mortality in this meta-analysis, the weight of the study was higher in the study by Duley et al. (79.2%) [13] than in that by Andersson et al. (20.8%) [14], possibly because of the large sample size. Both studies favored the experimental group (DCC) in terms of mortality. Heterogeneity was not present because the p-value was 0.44 and I² = 0%. The overall effect (Z) of the meta-analysis in the two studies in terms of mortality was 2.01 with a statistically significant p-value of 0.04. The pooled effect and 95% CI did not cross the line of no effect (Figure 2).

**Figure 3:**
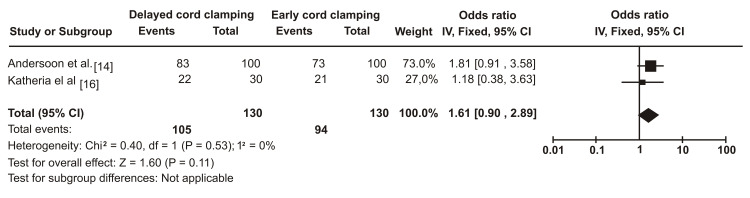
APGAR Score (>7) at 5 Mins among Infants With Delayed Cord Clamping (DCC) Relative to Early Cord Clamping (ECC) APGAR: appearance, pulse, grimace, activity, and respiration

When the 5-minute APGAR (appearance, pulse, grimace, activity, and respiration) value was compared in this meta-analysis, the weight of the study is higher in Andersson et al. (73%) [14] than in Katheria et al. (27%) [16], possibly due to the sample size. Heterogeneity was not present as the Chi² value was 0.40, the p-value was 0.53, and the I² value was 0%. The overall effect (Z) of meta-analysis in the two studies according to APGAR score was 1.60 with a p-value of 0.11. The pooled effect and 95% CI crossed the line with no effect (0.9-2.89) (Figure 3).

From the above meta-analysis, we noted that mortality was lower in the DCC group compared to the early cord clamping group. However, there was no statistically significant difference in the 5-minute APGAR scores in both groups.

Discussion

Both preterm and term infants may benefit from cord clamping. It lessens physiological anemia by raising hemoglobin levels at birth. The benefits of transitional circulation are greater for preterm infants thus the possibility of intraventricular bleeding is decreased. The mother is not at risk of blood loss. Several observational studies and clinical trials have indicated that intact cord resuscitation produces positive results. Even though there is evidence most developed and underdeveloped countries do not adopt. This review was conducted to emphasize the significance of intact cord resuscitation.

Out of the four trials included, two were from Nepal, one was from the USA, and one was from the UK. All studies were randomized control trials and included neonates who required resuscitation in accordance with the Hybrid Big Bang (HBB) algorithm (Table 1). Only Duley et al.'s (2017) study included newborns if their GE was less than 33 weeks; other studies included neonates of GE >33 weeks, and they required resuscitation in accordance with the HBB algorithm, which is defined as no breathing within 30 seconds after delivery despite complete drying and further stimulation [13]. Andersson et al.'s (2019) study found a significant impact of delayed clamping > 180 s. They noted significantly higher 5.0%, 10% DC-oxygen saturation, and APGAR scores, respectively.

In the intact cord group, the absolute risk reduction was 56%, i.e., they had SpO2 > 90%, whereas none had this state in the early cord clamping group [14]. Other studies did not find any statistically significant impact of delayed clamping on resuscitation. Mortality was studied by Duley et al. (2017), who found a significant (p = 0.04) impact of DCC, whereas Andersson et al. (2019) found it insignificant (p = 0.07) (Table 2) [13,14]. One study by Isacson et al. (2021) focused on neurodevelopmental outcomes and noted improvements after two years of birth, although there was no significant difference in the language-cognitive motor and socio-emotional domains [15]. Katharia et al. (2017) did not note any significant improvement in newborns with intact cords for 5 minutes; however, the 5-minute DCC group had greater blood pressure and cerebral oxygenation [16].

In a comprehensive review on the subject of DCC in preterm infants, Fogarty M noted that mortality would be reduced [17]. Only premature infants were included in Chapman J's systematic study, [18] and his findings on hematocrit and intraventricular hemorrhage supported DCC. Garg BD et al. matched late clamping with merely necrotizing enterocolitis [19].

In our review, the overall effect (Z) of meta-analysis in the two studies related to mortality was 2.01, with a statistically significant p-value of 0.04. The overall effect (Z) of meta-analysis in the two studies related to APGAR score was 1.60 with a statistically insignificant value of 0.11 (p-value). This is due to the small sample size in the study by Katharia et al., although this study included term babies, which favors the APGAR score because, as mentioned earlier, a small sample size can alter the results. Both studies usually have enough sample size in the mortality group. Because of that, there are more chances for a justifiable outcome for DCC.

As noted above, because DCC is a natural process, most of the data showed that DCC is a favorable strategy, and more trials are needed to reinforce the process. Because of fewer trials, our study power was poor, this is the major limitation of our study.

## Conclusions

The majority of available studies are in favor of intact cord resuscitation in neonates. Most of the studies are in developed countries where technology and manpower are higher. We need to reassess in low- and middle-income countries where the burden is high with low manpower. However, as per our review, intact cord resuscitation is beneficial to the newborn. A large number of randomized trials were needed to strengthen the study, particularly in preterm infants.
